# Major Adverse Cardiovascular Events in Treated Periodontitis: A Population-Based Follow-Up Study from Taiwan

**DOI:** 10.1371/journal.pone.0130807

**Published:** 2015-06-26

**Authors:** Shing-Hsien Chou, Ying-Chang Tung, Yu-Sheng Lin, Lung-Sheng Wu, Chia-Pin Lin, Eric Jein-Wein Liou, Chee-Jen Chang, Suefang Kung, Pao-Hsien Chu

**Affiliations:** 1 Division of Cardiology, Department of Internal Medicine, Chang Gung Memorial Hospital, Taipei, Taiwan; 2 Clinical Informatics and Medical Statistics Research Center, Chang Gung Memorial Hospital, Taipei, Taiwan; 3 Department of Orthodontics and Craniofacial Dentistry, Chang Gung Memorial Hospital, Taipei, Taiwan; 4 Division of Periodontology, Chang Gung Memorial Hospital, Taipei, Taiwan; 5 Cheers Dental Clinic, New Taipei, Taiwan; 6 Healthcare Center, Chang Gung Memorial Hospital, Taipei, Taiwan; 7 Heart Failure Center, Chang Gung Memorial Hospital, Chang Gung University College of Medicine, Taipei, Taiwan; National Cardiovascular Center Hospital, JAPAN

## Abstract

**Background:**

The aim of the present study was to identify the long-term major adverse cardiovascular events (MACE) in treated periodontitis patients in Taiwan.

**Methods:**

From the National Health Insurance Research Database (2001-2010), adult patients (≥ 18 years) with treated periodontitis were identified. Comparison was made between patients with mild form and severe form of treated periodontitis after propensity score matching. The primary end point was the incidence of MACE.

**Results:**

A total of 32,504 adult patients with treated periodontitis were identified between 2001 and 2010. After propensity score matching, 27,146 patients were preserved for comparison, including 13,573 patients with mild form and 13,573 patients with severe form of treated periodontitis. During follow-up, 728 individuals in mild treated periodontitis group and 1,206 individuals in severe treated periodontitis group had at least 1 MACE event. After adjustment for gender, hyperlipidemia, hypertension and diabetes mellitus, severe treated periodontitis was associated with a mildly but significantly increased risk of MACE among older patients > 60 years of age (incidence rate ratio, 1.26; 95% confidence interval, 1.08–1.46). No association was found among younger patients ≤ 60 years of age.

**Conclusions:**

Severe form of treated periodontitis was associated with an increased risk of MACE among older Taiwanese patients, but not among younger Taiwanese patients. We should put more efforts on the improvement of periodontal health to prevent further MACE.

## Background

Cardiovascular disease (CVD) is a major cause of morbidity and mortality worldwide. In the year 2010, CVD accounted for 31.9% (787,650) of all 2,468,435 deaths in the United States[1]. In Taiwan, cardiac diseases and cerebrovascular diseases were the second and third most common causes of death following malignant neoplasms, comprising 11.1% and 7.2% of the total deaths in the year 2012[2]. Diabetes mellitus, hypertension, dyslipidemia, cigarette smoking, and obesity are well-established risk factors for CVD. Nevertheless, the incidence of CVD remained high despite of efforts on managing these modifiable comorbidities. Furthermore, a portion of cases with CVD cannot be sufficiently explained by these risk factors. Recently inflammation has emerged to play a fundamental role in the pathogenesis of atherosclerosis[3–8], and markers of low-grade inflammation have been consistently associated with a higher risk of cardiovascular disease[4, 8, 9]. In the past two decades, periodontitis has attracted considerable attention as a possible novel risk factor for CVD.

Periodontitis is a chronic multifactorial inflammatory disease caused by microorganisms and characterized by progressive destruction of the tooth supporting apparatus leading to tooth loss[10]. Periodontitis increases systemic inflammation stimulated by bacteria and their products, induces cross-reactive antibodies that promote inflammation and interact with the atheroma, and alters lipid metabolism with consequent increases in pro-inflammatory lipid classes and subclasses[11]. These common traits of periodontitis support the biologically plausible link between CVD and periodontitis.

The epidemiologic evidences of association between periodontitis and certain specific cardiovascular diseases, such as coronary heart disease[12–20] and stroke[13, 16, 19–26], have been elucidated since 1990s. However, there is still insufficient evidence to support such association between periodontitis and all types of MACE and in all groups of population[27–30]. Larger scaled long-term epidemiologic studies in different ethnic groups are also missing. Therefore, the aim of the current study was to clarify the impacts of treated periodontitis on all MACE by analyzing a large-scale, population-based longitudinal data in an Asian population from National Health Insurance (NHI) claims records in Taiwan. Dose effects of periodontitis on each MACE in different age groups are also analyzed.

## Methods

### Data source and the study cohort

This is a nationwide population-based retrospective cohort study using the claims database of the National Health Insurance Research Database (NHIRD) from 2001–2010 in Taiwan. Currently, the National Health Insurance program covers more than 99% of the population of Taiwan[31], and the NHIRD provides medical claims, registration, and reimbursement data[32–34].

**Fig 1:**
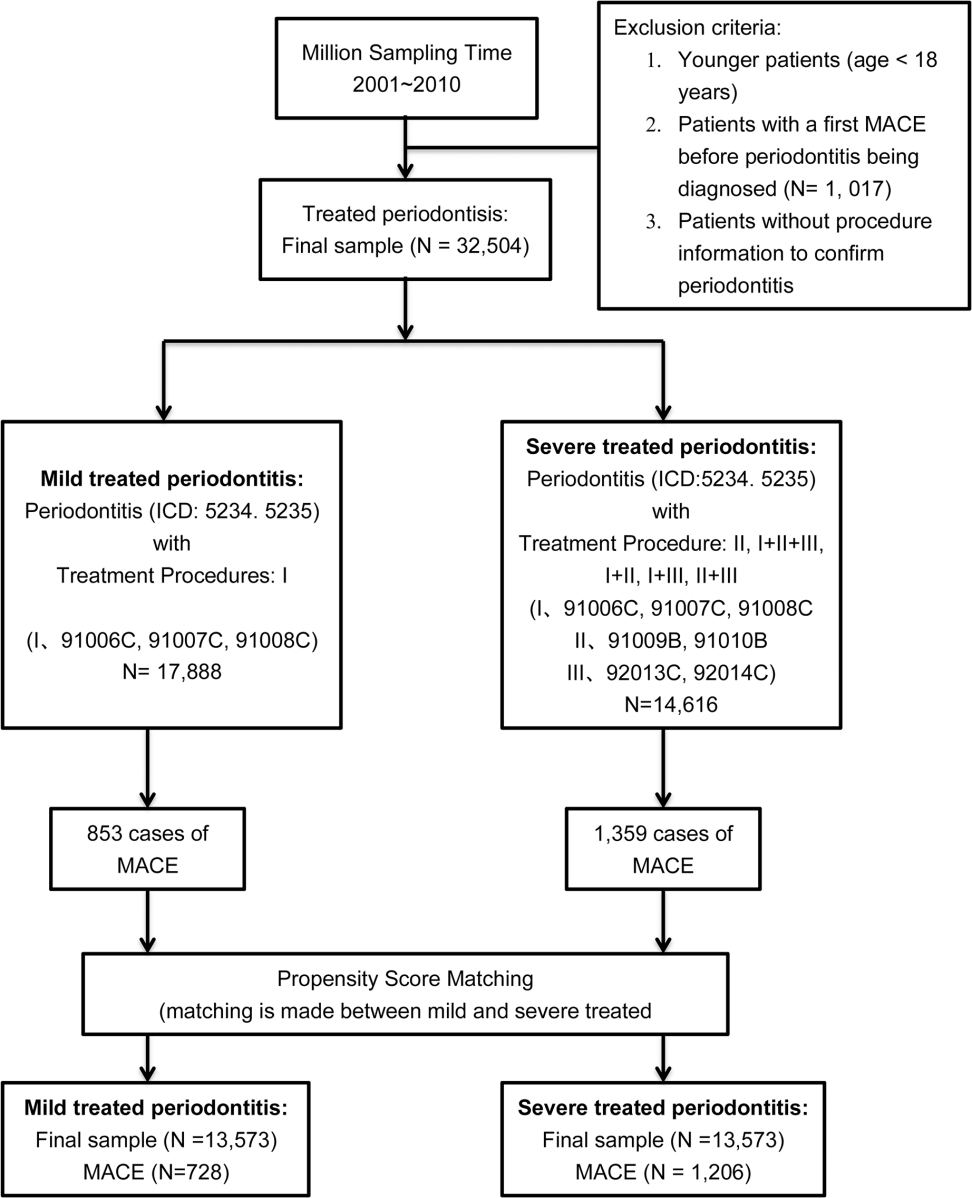
Selection of study subjects and identification of major adverse cardiovascular events (MACE) in treated periodontitis in Taiwan from 2001 to 2010.

During 2001–2010, 32,504 adult patients (aged ≥ 18 years) with treated periodontitis were identified from the random sample of 1,000,000 insurants of the National Health Insurance program (a subset of the NHIRD). Younger patients (aged <18 years), the patients with a first MACE before periodontitis being diagnosed, and the patients without procedure information to confirm them were excluded (Fig 1). Diagnostic information was based on the International Classification of Diseases, Ninth Revision, Clinical Modification (ICD-9-CM). Diagnostic codes of periodontitis (ICD-9 5234 and 5235) and procedure codes (91006C, 91007C, 91008C, 91009B, 91010B, 92013C, and 92014C) were used. The procedure codes were grouped into I (91006C, 91007C or 91008C; subgingival curettage of full mouth, half arch or less than 3 teeth respectively), II (91009B, 91010B; periodontal flap operation of less than 3 teeth or 4 to 6 teeth respectively) and III (92013C, 92014C; simple extraction or complicated extraction respectively). Accordingly, mild form of treated periodontitis was defined by a diagnostic code and a procedure code of group I; severe form was defined by a diagnostic code and any combination of these procedure codes except solely group I or group III.

Informed consent was waived as the database analysis used de-identified secondary data. The study was approved by the ethical committee (Institutional Review Board) of Chang Gung Memorial Hospital (#98-4060B).

### Identification of MACE cases

The primary outcome of this study was newly diagnosed MACE during the study period. Newly diagnosed cases of MACE were identified by a diagnosis of MACE (myocardial infarction, MI: ICD-9-CM code: 410–410.9; percutaneous coronary intervention, PCI: operation code: 36.0–36.03, 36.05–36.09; coronary artery bypass grafting, CABG: operation code: 36.1–36.99, V45.8; heart failure, HF: ICD-9-CM code: 428.0–428.10; cerebrovascular accident, CVA: ICD-9-CM code: 430–437; malignant dysrhythmia, MD: IDC-9-CM code 426.0, 426.12–426.13, 426.51, 426.52, 426.54, 427.1, 427.4, 427.41, 427.42, and 427.5; thrombolysis, Throm: operation code: 36.0–36.9; cardiac shock, CS: ICD-9-CM code 785.51).^[^ ^32^ ^,^ ^33^ ^]^

### Statistical analysis

Propensity score matching method was performed in which matching was taken place for those patients with mild treated periodontitis to the patients with severe treated periodontitis by several baseline variables, including age, gender, hyperlipidemia (ICD-9-CM code 272), hypertension (ICD-9-CM code 401–405, 4372, 36211) and diabetes mellitus (ICD-9-CM code 250, 3572, 36201, 36202, 36641). Group characteristics at baseline were compared using χ^2^ tests.

The person-time for each participant was calculated from the date of confirmed periodontitis during 2001–2010 to the date of any type of MACE, death, insurance discontinuation, or December 31, 2010, whichever came first. Poisson regression models were used to estimate incident MACE. Competing risk method was used to estimate incidence rate ratio (IRR) and 95% confidence intervals (95% CI) for total MACE[35]. Cumulative incidence curves were also generated from the competing risk method. A *p* value less than 0.05 is considered to be statistically significant. Statistical analyses were performed using SAS software (version 9.2; SAS institute Inc., Cary, NC, USA) and R software (version 3.0.2; The r foundation for statistical computing).

## Results

Between 2001 and 2010, a total of 32,504 patients with treated periodontitis were identified, including 17,888 and 14,616 patients with mild form and severe form respectively (Fig 1). After 1:1 propensity score matching by age, gender, and three major risk factors (hyperlipidemia, hypertension, and diabetes mellitus), we enrolled 13,573 patients with mild treated periodontitis (mild TP) and 13,573 matched patients with severe treated periodontitis (severe TP) in this study. MACE occurred in 728 and 1,206 patients in the mild and severe TP cohorts. Detailed baseline demographic and clinical characteristics are shown in Table 1. After matching, the potentially confounding baseline characteristics did not differ significantly between the two cohorts. The mean age (and standard deviation) of the mild and severe TP cohort was 48.2 (SD, 11.8) and 48.7 (SD, 11.5) years, with mean follow-up period of 3.6 (SD, 2.6) and 5.7 (SD, 2.8) years. Male patients accounted for 52.6% in both cohorts, and a plurality of patients had hyperlipidemia (22.8%), hypertension (22.9%) or diabetes (14.3%). The majority of these patients were equal or less than 60 year-old (11,554 (85.1%) and 11,462 (84.4%) patients in mild and severe TP cohort). 2,019 (14.9%) and 2,111 (15.6%) patients in mild and severe TP cohort were more than 60 year-old, respectively.

**Table 1:** Demographic and clinical characteristics of the study population before and after propensity score matching.

		Before propensity score matching			After propensity score matching		
		Mild TP* (n = 17888)	Severe TP* (n = 14616)	p-value	Mild TP* (n = 13573)	Severe TP* (n = 13573)	p-value
		n (%)	n (%)		n (%)	n (%)	
Age Group				<0.0001			1.0000
(years)	<35	3769 (21.1)	1474 (10.1)		1474 (10.9)	1474 (10.9)	
	35–50	7728 (43.2)	6654 (45.5)		6353 (46.8)	6353 (46.8)	
	50–65	5039 (28.2)	5049 (34.5)		4512 (33.2)	4512 (33.2)	
	65–80	1239 (6.9)	1337 (9.2)		1140 (8.4)	1140 (8.4)	
	≥80	113 (0.6)	102 (0.7)		94 (0.7)	94 (0.7)	
Age 60 Group				<0.0001			0.1200
(years)	≤60	15640 (87.4)	12193 (83.4)		11554 (85.1)	11462 (84.4)	
	>60	2248 (12.6)	2423 (16.6)		2019 (14.9)	2111 (15.6)	
Sex				<0.0001			
	Male	8198 (45.8)	8108 (55.5)		7144 (52.6)	7144 (52.6)	1.0000
	Female	9690 (54.2)	6508 (44.5)		6429 (47.4)	6429 (47.4)	
Risk factor							
	Hyperlipidemia	3985 (22.3)	3161 (21.6)	0.1589	3089 (22.8)	3089 (22.8)	1.0000
	Hypertension	3683 (20.6)	3299 (22.6)	<0.0001	3113 (22.9)	3113 (22.9)	1.0000
	Diabetes mellitus	2327 (13.0)	2138 (14.6)	<0.0001	1940 (14.3)	1940 (14.3)	1.0000
Mean age (SD), years					48.2(11.8)	48.7(11.5)	0.0003
Mean follow up (SD), years					3.6(2.6)	5.7(2.8)	<0.0001

* TP, treated periodontitis; SD, standard deviation.

The incidence of MACE among different demographic and clinical groups by types of MACE after matching is shown in Table 2. In older patients (age > 60 years old), the incidence of total MACE reached 4,966 and 5,393 per 100,000 person-years in mild and severe TP group, respectively (P = 0.73). In younger patients (age ≤ 60 years old), the incidences were 1,062 and 1,075 per 100,000 person-years in mild and severe TP group (P = 0.75).

**Table 2:** The incidence of MACE among different demographic and clinical groups by types of MACE in treated periodontitis (TP) in Taiwan from 2001 to 2010.

≤60 years																			
		**MI**	** **	**PCI**	** **	**CABG**	** **	**HF**	** **	**CVA**	** **	**MD**	** **	**Throm**	** **	**CS**	** **	**MACE**	** **
Total		68	64.0	61	57.4	19	17.9	120	113.1	819	787.9	37	34.8	76	71.5	3	2.8	1103	1070.0
Sex																			
	Male	57	100.4	57	100.4	17	29.9	58	102.2	459	826.4	23	40.4	70	123.3	1	1.8	650	1182.7
	Female	11	22.2	4	8.1	2	4.0	62	125.6	360	743.8	14	28.3	6	12.1	2	4.0	453	941.4
Risk factor																			
	Hyperlipidemia	25	145.1	29	168.1	7	40.4	40	232.2	257	1553.3	11	63.6	35	203.0	0	0.0	360	2215.6
	Hypertension	36	223.1	27	166.9	12	74.0	48	297.8	263	1699.3	10	61.6	37	229.1	2	12.3	389	2577.2
	Diabetes mellitus	13	119.1	21	192.3	5	45.6	27	247.5	191	1836.9	8	73.1	26	238.4	0	0.0	261	2558.5
TP stage																			
	mild	16	38.8	21	48.5	8	19.4	46	111.8	332	821.6	9	21.8	26	63.1	0	0.0	427	1062.4
	severe	52	80.0	41	63.0	11	16.9	74	113.9	487	766.5	28	43.0	50	76.8	3	4.6	676	1074.9
>60 years																			
		**MI**	** **	**PCI**	** **	**CABG**	** **	**HF**	** **	**CVA**	** **	**MD**	** **	**Throm**	** **	**CS**	** **	**MACE**	** **
Total		32	172.6	54	292.6	14	75.3	158	868.5	564	3376.3	21	112.9	61	330.8	3	16.1	831	5229.9
Sex																			
	Male	21	217.1	39	406.0	10	103.0	94	993.3	299	3447.7	10	102.9	43	448.0	3	30.8	465	5694.1
	Female	11	124.0	15	169.5	4	45.0	64	733.3	265	3299.2	11	123.9	18	203.6	0	0.0	366	6205.4
Risk factor																			
	Hyperlipidemia	12	168.0	26	366.2	6	83.8	69	986.4	254	4005.3	7	97.6	29	408.8	2	27.9	373	6205.4
	Hypertension	24	243.7	37	377.5	11	111.1	108	1121.5	375	4311.0	16	161.6	42	428.9	2	20.1	564	6943.1
	Diabetes mellitus	13	246.4	22	420.4	7	132.3	71	1389.5	207	4419.3	9	170.0	25	478.4	0	0.0	324	7477.0
TP stage																			
	mild	15	219.9	15	220.0	8	116.8	56	831.3	206	3266.7	8	116.7	18	264.1	2	29.1	301	4965.7
	severe	17	145.0	39	335.1	6	51.1	102	890.5	358	3442.7	13	110.7	43	369.9	1	8.5	530	5392.9

Values are numbers of events (incidence rate, i.e., 100,000 patients per year); MACE, major adverse cardiovascular events; MI, myocardial infarct; PCI, percutaneous coronary intervention; CABG, coronary artery bypass grafting; HF, heart failure; CVA, cerebrovascular accident; MD, malignant dysrhythmia; Throm, thrombolysis; CS, cardiac shock.

In the overall 1,070 and 5,230 cases per 100,000 person-years of any MACE in younger and older patient groups, the majority consisted of stroke cases, including 788 and 3376 cases per 100,000 person-years, respectively. Heart failure, MI, PCI and thrombolysis were the next most common MACE. This distribution of individual MACE was similar among different gender, risk factors and TP groups.

**Fig 2:**
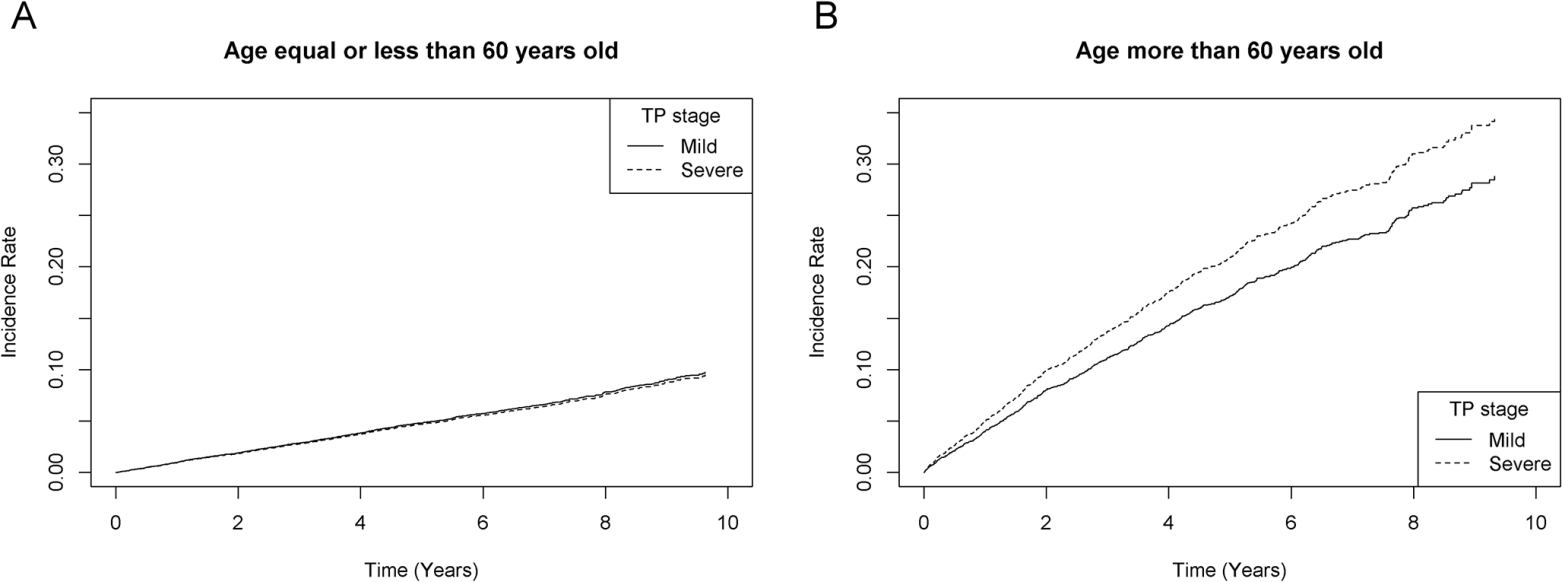
The cumulative incidence of major adverse cardiovascular events (MACE) associated with mild and severe treated periodontitis (TP). A. The cumulative incidence of MACE among younger patients (age ≤ 60 years old); B. The cumulative incidence of MACE among older patients (age > 60 years old).

Fig 2 shows the cumulative incidence of MACE associated with mild and severe TP groups. Among older patients, those with severe TP were associated with higher cumulative incidence of MACE than those with mild TP (P < 0.01). No association between TP severity and incidence of MACE was presented among younger patients (P = 0.37).

The results of multivariate competing risk regression model are summarized in Table 3. After adjusting for gender and three major risk factors, older patients with severe TP showed a mildly increased risk of MACE (incidence rate ratio: 1.26, 95% CI: 1.08–1.46) compared to those with mild TP. Among younger patients, the risk was not significantly different between mild and severe TP groups. Gray’s test showed the difference between mild and severe TP was significant for total MACE in older patients (P < 0.01), but insignificant in younger patients (P = 0.37).

**Table 3:** Incidence rate ratio (IRR) for MACE by multivariate competing risk regression model in treated periodontitis (TP) in Taiwan from 2001 to 2010.

MACE						
Age 60 Group	≤60 years			>60 years		
Effect	IRR*	95% CI	P-value	IRR*	95% CI	P-value
Sex Male vs Female	1.17	1.03–1.32	0.01	1.10	0.95–1.28	0.19
Hyperlipidemia	1.61	1.37–1.88	<0.01	1.11	0.95–1.31	0.19
Hypertension	2.30	1.99–2.67	<0.01	1.69	1.44–1.99	<0.01
Diabetes mellitus	1.65	1.39–1.95	<0.01	1.35	1.15–1.58	<0.01
TP stage Severe vs Mild	0.94	0.83–1.07	0.37	1.26	1.08–1.46	<0.01

*Incidence rate ratio by multivariate competing risk regression model was adjusted for sex, hyperlipidemia, hypertension, diabetes and TP stage; MACE, major adverse cardiovascular events; CI, confidence interval.

## Discussion

Abundant evidences from previous epidemiologic studies had demonstrated that adults with periodontal diseases (PD) had increased risk of coronary heart disease[12–20] and cerebrovascular disease[13, 16, 19–26] compared to the normal population. From a retrospective cohort study of 10,368 persons with 23-year follow-up by Morrison et al[14], those with severe gingivitis and edentulous status were found to have a 2.15 (95% CI: 1.25–3.72) and 1.90-fold (95% CI: 1.17–3.0) elevation in the risk of fatal coronary heart disease. Wu et al[21] found adults with gingivitis, periodontitis and edentulousness were 1.24 (95% CI: 0.74–2.08), 2.11 (95% CI: 1.30–3.42) and 1.41-fold (95% CI: 0.96–2.06) more likely to have ischemic stroke. On the contrary, considerable studies showed negative results of the association between PD and MACE[27–30]**.** A prospective study by Howell et al suggested that self-reported periodontitis is not an independent predictor for subsequent cardiovascular diseases in US physicians[28]. However, more recent review studies including one by an American Heart Association (AHA) working group analyzed 537 peer-reviewed publications, and concluded that periodontal disease is associated with atherosclerotic vascular disease independent of known confounding factors[19].

Our report is a large-scale and long-term Asian population-based study that demonstrated an age-dependent association between treated periodontitis and MACE. We found that patients with severe TP were more likely to develop MACE than mild TP in older group (> 60 year old), but not in younger group (≤ 60 year old). Older patients with severe TP had a 1.26-fold elevation in the incidence rate risk of any MACE (95% CI: 1.08–1.46) even after adjusting for gender, hypertension, hyperlipidemia and diabetes mellitus. Our study showed the possible dosage effect of periodontitis on MACE among older patients, which was consistent with previous studies[13, 22]. In a prospective cohort study of 1,147 men in 1996, Beck et al discovered that those with high alveolar bone loss had almost twice the incidence of fatal coronary heart disease, and three-fold risk of stroke as those with low bone loss[13]. They showed a biologic gradient between severity of exposure and occurrence of disease and also proposed that periodontal disease, as chronic Gram-negative infection, provides a biological burden of endotoxin (lipopolysaccharide) and inflammatory cytokines (especially TxA_2_, IL-1β, PGE_2_, and TNF-α) leading to initiation and exacerbation of atherogenesis and thromboembolic events.

The impact of periodontal therapy on risk modification of MACE had been investigated previously. Several studies[11, 19, 36–38] had reported improvement in inflammatory markers and endothelial function as measured by flow mediated dilation among adults with significant periodontitis who had undergone nonsurgical periodontal therapy. In a randomized study, Tonetti[36] et al suggested intensive periodontal treatment resulted in improvement of endothelial function 6 months after therapy. But to date, no study has proved such treatment to be associated with reduction of clinically significant cardiovascular events[19]. In our study, the adults with treated periodontitis were identified by combination of diagnostic codes and procedure codes based on the ICD-9-CM, which indicated periodontal therapy had been performed on them. Our study implied that the risk of MACE in older patients with severe treated periodontitis still elevated even after periodontal treatment.

Several mechanisms have been proposed as potential links between periodontitis and cardiovascular disease, including causal and noncausal pathways[17, 19]. In causal pathways, periodontitis is associated with increased systemic inflammatory markers, including C-reactive protein (CRP)[39–44] and fibrinogen[43, 45]. CRP concentration has continuous associations with the risk of coronary heart disease, ischemic stroke and vascular morality[4, 8]. Subjects with periodontitis may be more susceptible to atherogenesis or atheromatous plaque rupture through activation of systemic inflammation[46]. Secondly, cross-reactive antibodies between periodontal bacterial heat-shock protein (HSP) termed GroEL and host HSP60 have been demonstrated[47, 48]. In animal model of apolipoprotein-E-deficient (-/-) mice, immunizations with P. gingivalis enhanced atherosclerosis. Host HSP60 was detected in atherosclerotic lesions, and the lesion development was correlated with anti-GroEL antibody levels[49]. Thirdly, direct bacteremia and vascular infection by periodontal pathogens has been proposed[19]. Periodontal bacterial DNA[50–52] and viable pathogens[53] were detected in endarterectomy specimens of carotid or femoral arteries in some studies. However, the evidence was not consistent in other studies[54, 55]. Finally, periodontitis is associated with altered lipid profiles in favor of atherosclerosis. Clinical studies demonstrated increased total cholesterol, low-density lipoprotein (LDL), Triglyceride and decreased high-density lipoprotein (HDL) concentration in patients with periodontitis[56–58]. Periodontal treatment resulted in decreased total cholesterol, LDL and oxidized LDL in some studies[59, 60], but not in the study by Losche et al[61]. In non-causal pathway, many established risk factors are shared by periodontitis and cardiovascular disease, and could increase the susceptibility to both diseases[17, 19]. These include genetic factors[62], age, smoking, diabetes mellitus, male sex, overweight or obesity, education and socioeconomic status[27, 29]. In our study, the risk of cardiovascular disease in older patients with severe treated periodontitis still elevated even after matching of several confounding risk factors, including age, sex, hyperlipidemia, hypertension and diabetes. Accordingly, the observed association between these two disorders was unlikely to be completely explained by these confounding risk factors.

There are some limitations to the current study. First, the NHI claims data does not include education and socioeconomic status. This may influence the result of our study because education and socioeconomic status correlate with both cardiovascular disease[63] and periodontitis[27, 29]. However, in a prospective cohort study of 8032 adults by Hujoel et al, stratifying the study participants by socioeconomic class did not alter the hazard ratio for coronary heart disease associated with periodontitis[27]. Secondly, the history of smoking is not available in the NHI claims data. The possibility of its confounding effect is thus unable to be evaluated in our study. Even so, it has been shown in recent studies that periodontitis was associated with cardiovascular disease in never-smokers as well[23, 64, 65]. We believe that the conclusion of this study still provides valuable information about the possible association between periodontitis and MACE even the causative relationship is not established. This conclusion is partly supported by previous review study which stated that although the two disease entities share several common risk factors including smoking, age and diabetes mellitus, there is still enough evidence to support an association between periodontitis and atherosclerotic vascular disease independent of known confounders[19]. Thirdly, comparison of MACE rate between periodontitis and non-periodontitis individuals is not practicable based on our study design. Instead, comparison was done between individuals with mild and severe form of periodontitis. In our study, adults with periodontitis but without searching medical aid would be misclassified as non-periodontitis. Besides, adults with periodontitis diagnosed after MACE were excluded. This may reduce the actual MACE rate in periodontitis patients, because in a portion of these patients, periodontitis may occur before MACE but be diagnosed clinically after MACE. Last, although older adults with severe periodontitis still posed higher risk of MACE after periodontal treatment, our study is unlikely to illustrate whether periodontal treatment will reduce the risk.

Despite such limitations, NHI claims database provides valuable information for understanding the association between periodontitis and major cardiovascular events. This issue has attracted more attention lately given the high incidence of both diseases, and the great impact of MACE on the health of victims. These cardiovascular complications place a large burden on not only individuals but also the society and health-care system. In summary, older adults with severe treated periodontitis have a significantly higher risk of major cardiovascular events, especially MI, coronary intervention, heart failure and stroke. The risk remains increased even after the treatment of periodontitis. Our results suggest that there should be close and continuous monitoring of both periodontal and cardiovascular status for those with severe periodontitis. Furthermore, stringent efforts should be made early enough to improve periodontal health and to prevent the occurrence of severe periodontitis when aged. This can in turn reduce the modifiable risk factors of major cardiovascular events in the elderly.

## Conclusions

Severe form of treated periodontitis was associated with an increased risk of MACE among older Taiwanese patients, but not among younger Taiwanese patients. We should put more efforts on the improvement of periodontal health to prevent further MACE.
